# Supplementary material for: Optogenetic control of gut bacterial metabolism to promote longevity
Source: eLife. 2020 Dec 16;9:e56849. doi: 10.7554/eLife.56849 (PMC7744093; doi:10.7554/eLife.56849)
Supplement: Supplementary file 1. — (a) Table of plasmids used in this study. (b) Table of bacterial and worm strains used in this study (c) Statistical analysis of worm lifespan experiments [file elife-56849-supp1.docx]

**Supplementary File 1a | Table of plasmids used in this study**

| Name | Size (bp) | Resistance | Origin of replication | Description | Reference |
| --- | --- | --- | --- | --- | --- |
| pLH401 | 4,770 | C | ColE1 | Constitutive *ccaR* expression, P*_cpcG2_*_-172_:*sfgfp*, constitutive *mcherry* expression | This study |
| pLH405 | 4,730 | C | ColE1 | Constitutive *ccaR* expression, P*_cpcG2_*_-172_:*gfpmut3**, constitutive *mcherry* expression | This study |
| pLH407 | 2,650 | C | ColE1 | Constitutive *mcherry* expression | This study |
| pLH412 | 6,282 | S | p15A | Constitutive *ccaS(H534A)*, *ho1*, and *pcyA* expression | This study |
| pLH413 | 3,848 | C | ColE1 | Constitutive *ccaR(D51N)* expression, P*_cpcG2_*_-172_:*rcsA* | This study |
| pSR43.6 | 6,282 | S | p15A | Constitutive expression of *ccaS, ho1,* and *pcyA* | Schmidl et al, 2014 |
| pSR49.2 | 4,543 | S | p15A | Constitutive expression of *ccaS* | Schmidl et al. 2014 |
| pMVK201.2 | 3,848 | C | ColE1 | Constitutive expression of *ccaR*, P*_cpcG2_*_-172_:*rcsA* | This study |
| pMVK228 | 4,588 | S | p15A | Constitutive expression of *ccaS* | This study |

C: chloramphenicol, S: spectinomycin

# Supplementary File 1b | Table of bacterial and worm strains used in this study

| Strain | Plasmids | Background | Resistance | Description | References |
| --- | --- | --- | --- | --- | --- |
| BW25113 | None | BW25113 | None | Keio parent strain | Baba et al., 2006 |
| Δ*rcsA* | None | JW1935-1 | K | Low CA production | Baba et al., 2006 |
| Δ*lon* | None | JW0429-1 | K | High CA production | Baba et al., 2006 |
| LH01 | pLH401,pSR43.6 | JW1935-1 | C,S,K | **Fig. 1,** microscopy | This study |
| LH02 | pLH401, pSR49.2 | JW1935-1 | C,S,K | ΔPCB control for LH01 | This study |
| LH03 | pLH405, pSR43.6 | JW1935-1 | C,S,K | **Fig. 1**, cytometry | This study |
| LH04 | pLH405, pSR49.2 | JW1935-1 | C,S,K | ΔPCB control for LH03 | This study |
| LH05 | pLH407, pSR43.6 | JW1935-1 | C,S,K | mCherry-only control for cytometry | This study |
| LH06 | pMVK201.2,pLH412 | JW1935-1 | C,S,K | CcaS(H534A) control for MVK29 (**Fig. 3**) | This study |
| LH07 | pLH413, pSR43.6 | JW1935-1 | C,S,K | CcaR(D51N) control for MVK29 (**Fig. 3**) | This study |
| MVK29 | pMVK201.2,pSR43.6 | JW1935-1 | C,S,K | Green light-induced CA secretion (**Fig. 3**) | This study |
| MVK46 | pMVK201.2,pMVK228 | JW1935-1 | C,K | ΔPCB control for MVK29 (**Fig. 3**) | This study |

## K: kanamycin

| Name | Genotype | CGC  Strain ID | Description | References |
| --- | --- | --- | --- | --- |
| *zu391* | *glo-1*(zu391) X | JJ1271 | Used for optogenetic induction of GFP expression in the worm gut. Exhibits fewer intestinal granules, which enables lower host background fluorescence. (**Fig. 1**) | Hermann et al., 2005 |
| *zcIs17* | *zcIs17*[P*_ges-1_*::*mito-GFP*] | SJ4143 | Used for optogenetic induction of CA production in the worm gut. N-terminal 30 amino acids sequence of yeast TOM70 is fused with GFP (Mito-GFP) is used to observe mitochondrial morphology in the intestine. (**Fig. 3**) | Han et al., 2017 |
| *zcIs14* | *zcIs14[P_myo-3_::mito-GFP]* | SJ4103 | Used for examining the effect of optogenetic-induced CA on muscular mitochondria. N-terminal 30 amino acids sequence of yeast TOM70 is fused with GFP (Mito-GFP) is used to observe mitochondrial morphology in the muscle (**Fig.** **3**) | Han et al., 2017 |
| *MW2241* | *raxIs145[P_ges-1_::mito-RFP]* |  | Used for examining the effect of optogenetic-induced CA on intesintal mitochondria. N-terminal 55 amino acids sequence of worm tomm-20 is fused with GFP (Mito-GFP) is used to observe mitochondrial morphology in the intestine (**Fig.** **3**) | In this paper |
| *e2117* | *sqt-3*(e2117)  V | CB4121 | Temperature-sensitive strain used for longevity studies. (**Fig. 4**) | Han et al., 2017 |

**Supplementary File 1c | Statistical analysis of worm lifespan experiments**

**
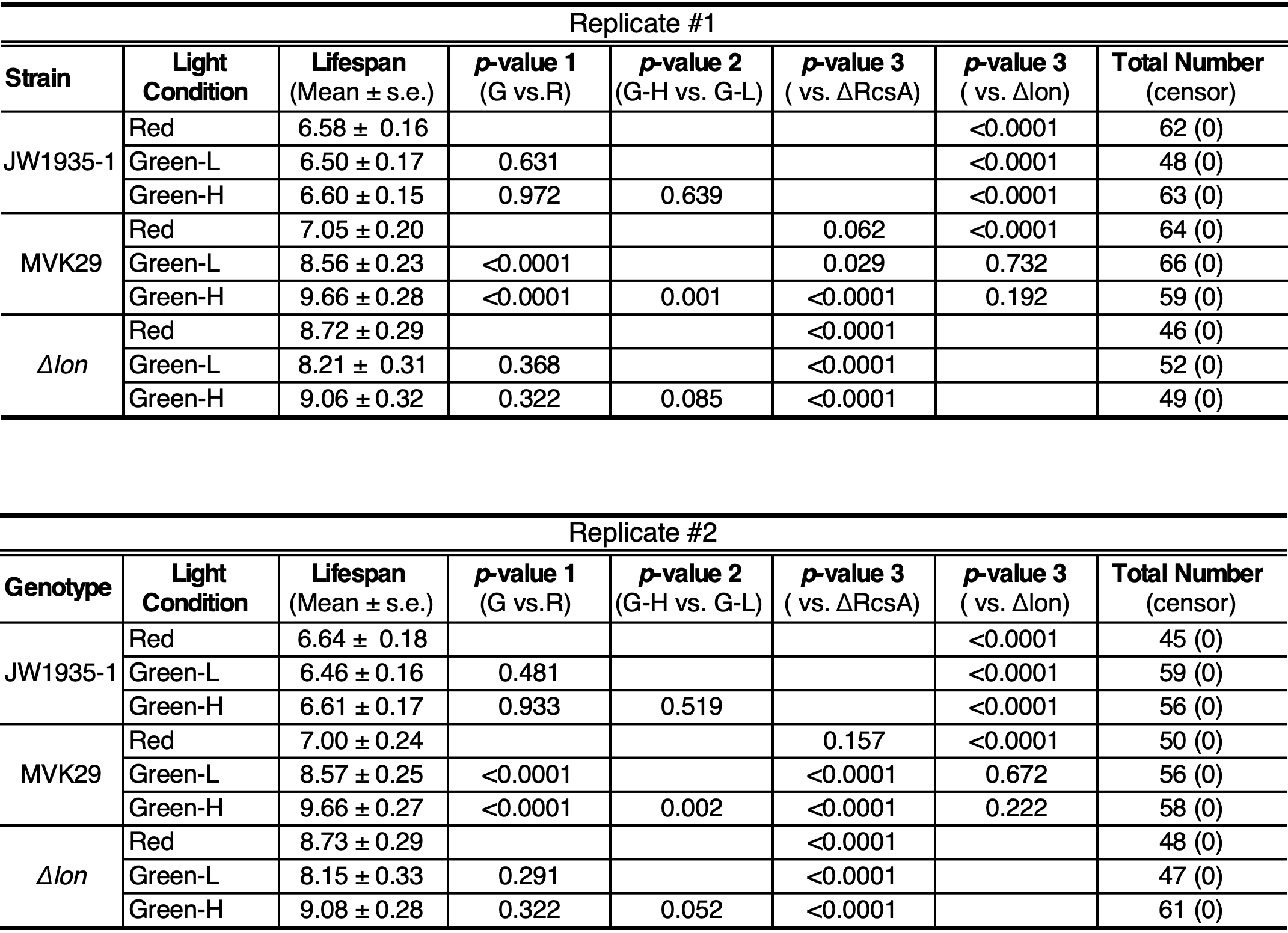
**

Censor worms are those that are lost or exhibit body bursting
